# Diversity of CRESS DNA Viruses in Squamates Recapitulates Hosts Dietary and Environmental Sources of Exposure

**DOI:** 10.1128/spectrum.00780-22

**Published:** 2022-05-26

**Authors:** Paolo Capozza, Gianvito Lanave, Georgia Diakoudi, Francesco Pellegrini, Roberta Cardone, Violetta Iris Vasinioti, Nicola Decaro, Gabriella Elia, Cristiana Catella, Alberto Alberti, Krisztián Bányai, Jairo Alfonso Mendoza-Roldan, Domenico Otranto, Canio Buonavoglia, Vito Martella

**Affiliations:** a Department of Veterinary Medicine, University of Bari, Valenzano, Italy; b Department of Veterinary Medicine, University of Sassari, Sassari, Italy; c Veterinary Medical Research Institute, Budapest, Hungary; d University of Veterinary Medicine, Budapest, Hungary; U.S. Food and Drug Administration

**Keywords:** reptiles, zoonosis, circovirus, cyclovirus

## Abstract

Replication-associated protein (Rep)-encoding single-stranded (CRESS) DNA viruses comprise viruses with covalently closed, circular, single-stranded DNA (ssDNA) genomes, and are considered the smallest known autonomously replicating, capsid-encoding animal pathogens. CRESS DNA viruses (phylum *Cressdnaviricota*) encompass several viral families including *Circoviridae*. Circoviruses are classified into two genera, *Circovirus* and *Cyclovirus*, and they are known to cause fatal diseases in birds and pigs. Circoviruses have also been identified in human stools, blood, and cerebrospinal fluid (CSF), as well as in various wild and domestic vertebrates, including reptiles. The synanthropic presence of Squamata reptiles has increased in the last century due to the anthropic pressure, which has shifted forested animal behavior to an urban and peri-urban adaptation. In this paper, we explored the diversity of CRESS DNA viruses in Squamata reptiles from different Italian areas representative of the Mediterranean basin. CRESS DNA viruses were detected in 31.7% (33/104) of sampled lizards and geckoes. Different CRESS DNA viruses likely reflected dietary composition or environmental contamination and included avian-like (*n* = 3), dog (*n* = 4), bat-like (*n* = 1), goat-like (*n* = 1), rodent-like (*n* = 4), and insect-like (*n* = 2) viruses. Rep sequences of at least two types of human-associated cycloviruses (CyV) were identified consistently, regardless of geographic location, namely, TN9-like (*n* = 11) and TN12-like (*n* = 6). A third human-associated CyV, TN25-like, was detected in a single sample. The complete genome of human-like CyVs, of a rodent-like, insect-like, and of a bat-like virus were generated. Collectively, the results recapitulate hosts dietary and environmental sources of exposure and may suggest unexpected ecological niches for some CRESS DNA viruses.

**IMPORTANCE** CRESS DNA viruses are significant pathogens of birds and pigs and have been detected repeatedly in human samples (stools, serum, and cerebrospinal fluid), both from healthy individuals and from patients with neurological disease, eliciting in 2013 a risk assessment by the European Centre for Disease Prevention and Control (ECDC). Sequences of CRESS DNA viruses previously reported in humans (TN9, TN12, and TN25), and detected in different animal species (e.g., birds, dogs, and bats) were herein detected in fecal samples of synanthropic squamates (geckos and lizards). The complete genome sequence of six viruses was generated. This study extends the information on the genetic diversity and ecology of CRESS DNA viruses. Because geckos and lizards are synanthropic animals, a role in sustaining CRESS DNA virus circulation and increasing viral pressure in the environment is postulated.

## INTRODUCTION

The family *Circoviridae* includes the smallest (10 to 20 nm) autonomously replicating animal viral pathogens. Circoviruses are non-enveloped and possess an icosahedral capsid and a circular, covalently closed, single-stranded DNA (ssDNA) genome, ranging from 1.7 to 2.1 kb. Genome has ambisense organization and contain at least two major (>600 nt) open reading frames (ORFs), encoding the replication-associated protein (Rep) and the capsid protein (Cap) (1–8). Advances in molecular diagnostics and metagenomic protocols have quickly generated data on circoviruses and other related rep-encoding circular ssDNA viruses (CRESS DNA), unveiling a large genetic diversity and ubiquitous distribution in various ecosystems (9–12). CRESS DNA viruses with different genome lengths and organization have been identified in environmental specimens, such as soil, sewage, and water, posing a challenge for their classification and nomenclature (13–15). These viruses has been recently grouped within the phylum *Cressdnaviricota*, thereby unifying at the mega-taxonomic level all officially recognized CRESS DNA virus families, including *Circoviridae* (16).

Circoviruses have been classified into two distinct genera. The genus *Circovirus* (CV) includes avian (17) and mammalian CVs while members of the genus *Cyclovirus* (CyV) have been found both in vertebrates and invertebrates (7, 8, 18, 19).

Recently, a series of novel CV and CyV have been detected in many host tissues and body fluids (e.g., stool, skin, liver, cerebrospinal fluid, blood, and nasopharyngeal aspirate samples) of several mammalian species (including beef, goat, camel, sheep, chimpanzee, rodent, dog, mink, bat, and humans) (14, 20–28), birds (12, 21, 28–36), fishes (37–39), insects (13, 17, 40–42), and reptiles (9, 43–45).

CV infections have relevance in veterinary medicine for causing severe diseases in birds and pigs. For instance, avian CV infection is associated with a variety of symptoms, including developmental abnormalities, lymphoid depletion, and immunosuppression (12, 46). In pigs, four different CVs have been identified, namely, porcine circovirus (PCV) 1 to 4 (47, 48). Only PCV2 is considered pathogenic as it has been associated with post-weaning multisystem wasting syndrome (PMWS), reproductive disorders (49), porcine respiratory disease complex (PRDC) (50), enteric disease (51), and dermatitis and nephropathy syndrome (PDNS) (48, 52) with comprehensive vaccination schemes being in place (47, 48).

Diverse CRESS DNA viruses have been recovered from human samples, including feces, cerebrospinal fluid, blood, serum, and respiratory secretions, of patients with central nervous system signs, lower respiratory tract infections, gastro-enteritis, and from overtly healthy individuals. The discovery of CRESS DNA viruses in human patients with neurological signs has raised concerns on the potential risks posed by these viruses to human health (21, 26, 53–56).

Information on CRESS DNA viruses in reptiles is still limited and there is scarce information regarding their diffusion, genetic diversity, and eventually whether and to which extent CRESS DNA viruses of reptiles may infect other animal species, or vice versa. Epidemiological investigations have shown that interspecies circulation of CRESS DNA viruses might not be uncommon (21, 22, 57). Due to the anthropic pressure (i.e., urbanization, habitat lost, and deforestation), forested animals have changed behavior and habitat use, adapting to urban and peri-urban areas, including Squamata reptiles such as snakes and lizards. Also, the use of reptiles as non-conventional pets (58), has increased the interactions between reptiles and humans generating additional zoonotic risks (58–60). Synanthropic reptiles in the Mediterranean basin are mainly represented by geckoes (i.e., *Tarentola* spp., *Hemidactylus* spp.) and lacertid lizards (i.e., *Podarcis* spp.). The change of behavior and use of habitat by these animals could enact the appearance of unforeseen epidemiological links, and this warrants investigations to explore the virome of these synanthropic animals. In this study we investigate circoviruses in different squamates species from urbane/peri-urban areas.

## RESULTS

### Identification of rep-like sequence in stool sample of Squamates and human sera.

A total of 104 stool samples from different reptile species, including the Italian wall lizard (*Podarcis siculus*, *n* = 66), the Maltese Wall Lizard (*Podarcis filfolensis*, *n* = 6), the Moorish ecko (*Tarentola mauritanica*, *n* = 13), the Western Whip Snake (*Hierophis carbonarius*, *n* = 2), the Hermann’s tortoise (*Testudo hermanii*, *n* = 2), the Ocellated Skink (*Chalcides ocellatus*, *n* = 12), and the Ball Python (*Python regius; n* = 3), were screened using a pan-circovirus degenerate nested PCR protocol (nPCR) (21). Most animals were adult (sexually mature) (77.8%, *n* = 81/104), male (60.5%, *n* = 63/104), and were collected from Apulia region, Italy (58.6%, *n* = 61/104). All tested animals either lived in urban/peri-urban areas or were housed as pets. A fragment of about 400 nt of the rep-like sequence was successfully amplified from 31.7% (*n* = 33/104) stool samples collected in the Italian regions of Apulia (29.5%, *n* = 18/61), Sicily (38.4%, *n* = 15/39), and Calabria (0%; *n* = 0/4) by nPCR (see Table S1 and Figure S1 in the supplemental material).

**FIG 1:**
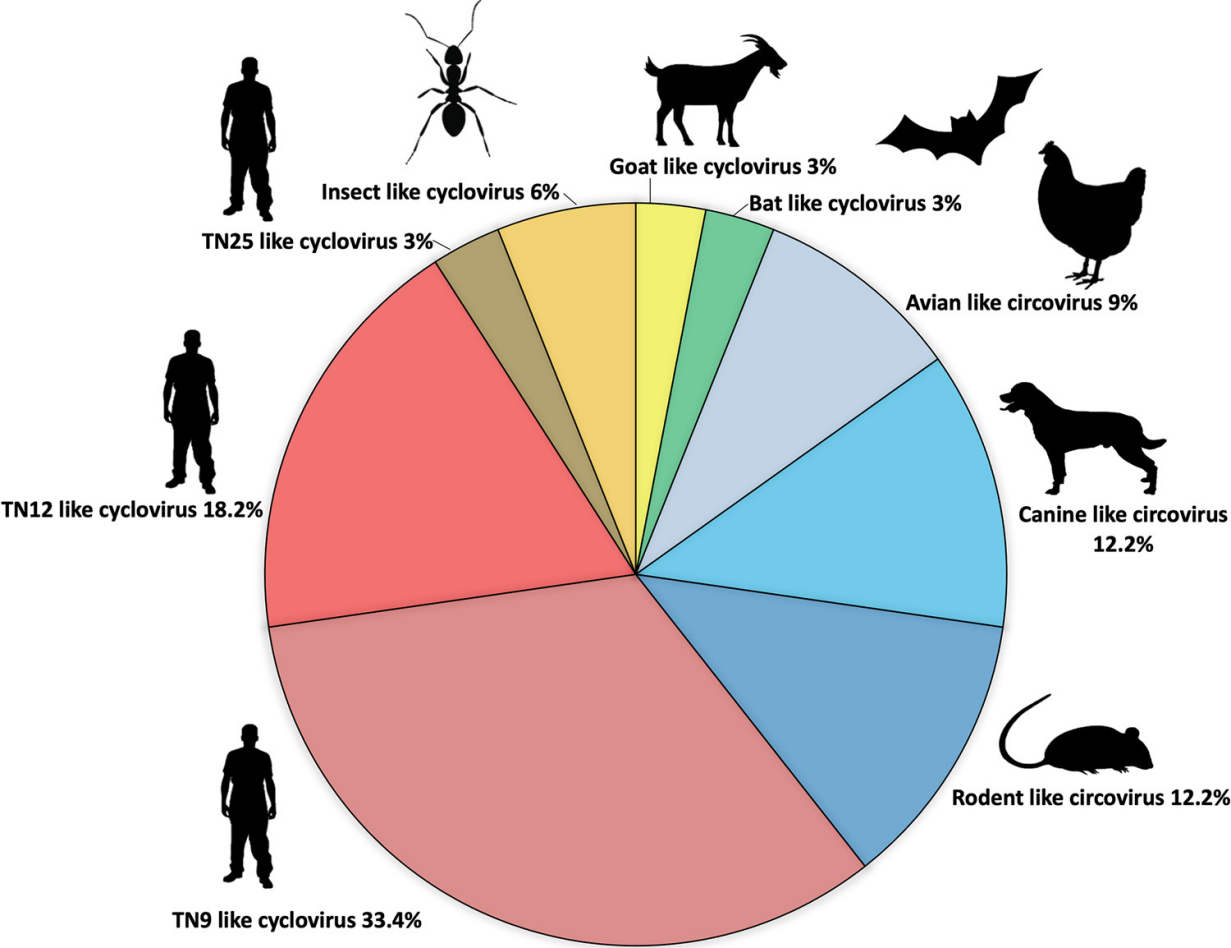
Results of genetic characterization of the circoviruses identified from Squamata reptiles based on partial Rep sequences. CV, circovirus; CyV, cyclovirus.

Sequences obtained from second-round PCR products were analyzed by nucleotide Basic Local Alignment Search Tool (BLAST, https://blast.ncbi.nlm.nih.gov/Blast.cgi), and a variety of rep-like sequences were identified (Fig. 1). Of these, three (9%) rep-like sequence, found in Italian wall lizard, shared 71% to 94% nucleotide (nt) identity with avian-like CVs. Four (12.2%) canine-like CVs (95% to 98% nt identity) were detected in the Italian wall lizard and the Moorish Gecko while four (12.2%) rodent-like CVs (69.5% to 70.3% nt identity) were detected in the Italian wall lizard and the Ocellated Skink. A stool sample from an Italian wall lizard tested positive to a bat-like CyV (86% nt identity). Moreover, the sequences of two (6%) insect-like CyV (72% nt identity) and one (3%) goat-like CyV (65% nt identity) were identified in Italian wall lizards. Rep sequences of at least two types of human-associated CyVs (HuA-CyV), TN9-like and TN12-like, were consistently identified, regardless of their geographic origin and reptile species. TN9-like HuA-CyV (97% to 98.5% nt identity in the partial Rep sequences) was detected in 11/33 (33.4%) animals, including the Italian wall lizard and the Western Whip Snake. TN12-like HuA-CyV (96.2% to 100% nt identity) was detected in 6/33 (18.2%) animals, including the Maltese Wall Lizard and the Ocellated Skink. A sample from the Italian wall lizard contained the Rep sequence of the TN25-like HuA-CyV (96% nt identity).

No significant statistical association was found between positivity to CRESS DNA virus and reptile species, sexual maturity, gender, and sampling site.

CRESS DNA viruses were not detected in the human sera (*n* = 101) collected from Pelagian islands.

### Genome characterization and phylogenetic analysis.

**FIG 2:**
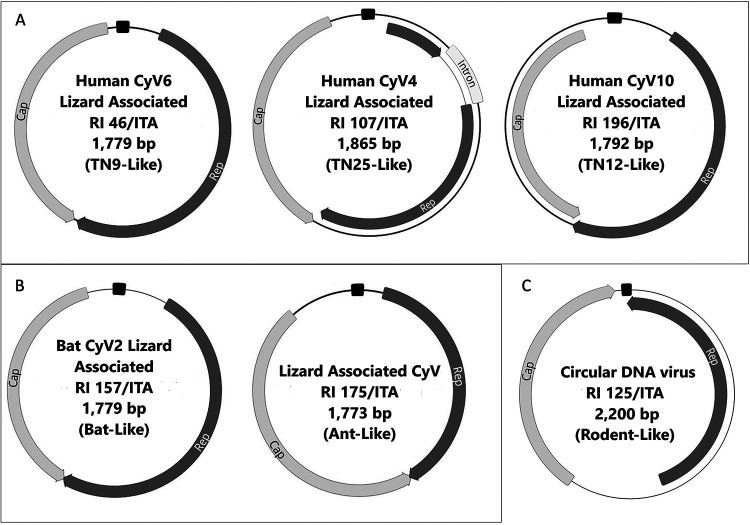
Genome schematic organizations of the Squamata reptile-associated viruses sequenced in this study. The cyclovirus strains RI 46/ITA, RI 107/ITA, and RI 196/ITA, classified as human-associated cycloviruses (HuACyV) 6, 4, and 10, respectively, are shown in panel A. The strain RI 157/ITA, classified as bat cyclovirus 2, and the unclassified ant-like cyclovirus RI 175/ITA, are shown in panel B. The unclassified rodent-like circular DNA virus RI 125/ITA is shown in panel C. The major ORFs, encoding the putative replication-associated protein (Rep) and the putative capsid protein (Cap) are shown (arrows). The location of the stem-loop structure is indicated (black rectangle). In brackets the alternative terminology used in the text of this manuscript is also reported.

**FIG 3:**
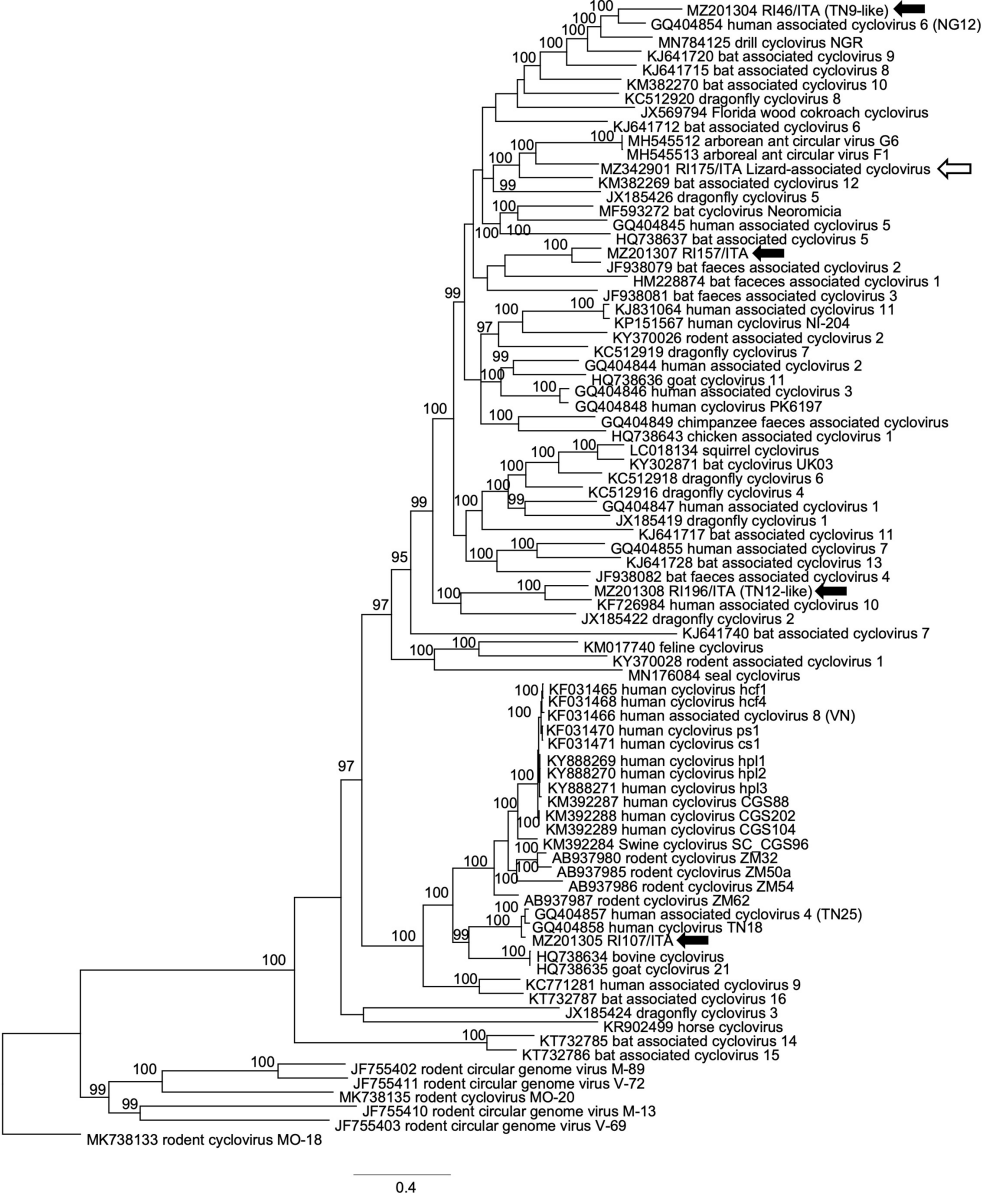
Phylogenetic tree based on the genome sequence of cycloviruses (CyVs). The sequences generated in this study were aligned with a selection of cognate sequences retrieved from GenBank following the updated (2021) ICTV outlines. Posterior output of the tree was derived from Bayesian inference using four chains run for >1 million generations, a general time-reversible model, a gamma distribution of rate variation across sites, and a subsampling frequency of 1,000. Posterior probability values >95 are indicated on the tree nodes. Black arrows indicate the CyV strains identified in this study. The white arrow indicates the candidate species, tentatively named lizard-associated CyV (LiACyV). Scale bar indicates nucleotide substitutions per site.

**FIG 4:**
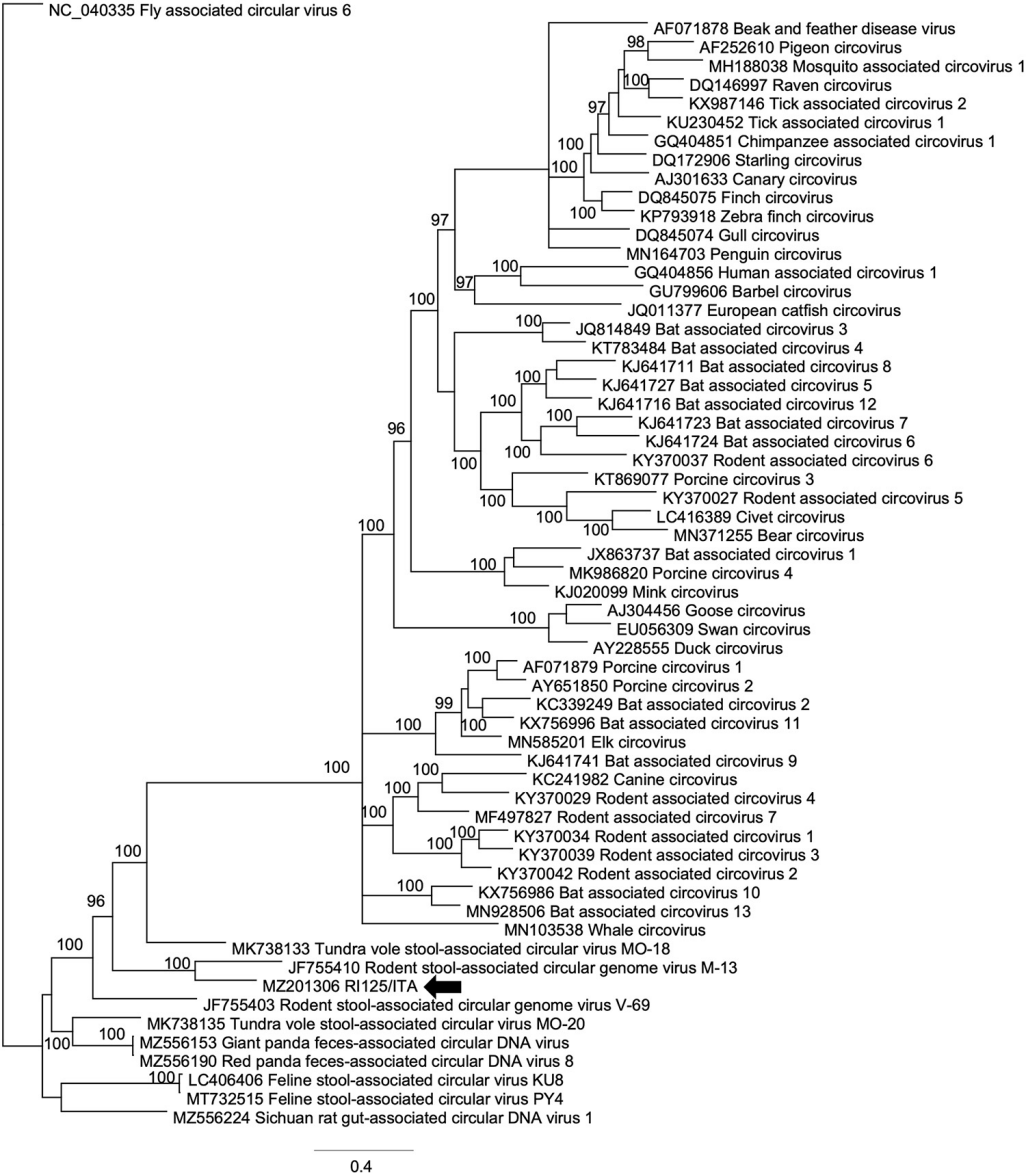
Phylogenetic tree based on the genome sequence of the circoviruses (CVs). The sequence generated in this study was aligned with a selection of cognate sequences retrieved from GenBank following the updated (2021) ICTV outlines. Posterior output of the tree was derived from Bayesian inference using four chains run for >1 million generations, a general time-reversible model, a gamma distribution of rate variation across sites, and a subsampling frequency of 1,000. Posterior probability values >95 are indicated on the tree nodes. The black arrow indicates the novel unclassified CRESS DNA virus detected in this study. Scale bar indicates nucleotide substitutions per site.

After enrichment of circular DNA by applying rolling cycle amplification (RCA) on selected samples representative of different sequence types (TN9-like, TN12-like, and TN25-like HuA-CyVs, rodent-like virus, arboreal ant-like CyV, and bat-like CyV), a total of six complete genomes were generated and their features are summarized in Table 1. For genome sequencing, an inverse PCR strategy was used, designing primers on the partial Rep sequences generated in second-round consensus PCR (Table S2). The ORFs and promoter regions conserved in circovirus genomes were mapped, reconstructing the genome organization of the six viruses (Fig. 2). Phylogenetic analyses (Fig. 3 and 4) were therefore carried out using the full genome sequences generated in this study and a selection of sequences extracted from the GenBank databases.

**TABLE 1:** Genomic features of complete genomes of circoviruses sequenced in this study^*a*^

Sample ID	Accession	Size (nt)	Putative rep		Putative cap		5′ intergenic region (nt)	3′ intergenic region (nt)	Loop motif (5′ to 3′)	Nt identity to		Originating reptile species
			nt	aa	nt	aa				ICTV^*b*^ species (accession nr.)	nt identity (%)	
RI46/ITA	MZ201304	1,779	915	305	708	236	148	8	TAATACTAT	HuA-CyV6 (GQ404854)	83.6	*Podacris siculus*
RI107/ITA	MZ201305	1,865	861	287	669	223	160	6	GTAATACTA	HuA-CyV4 (GQ404857)	95.9	*Podacris siculus*
RI196/ITA	MZ201308	1,792	837	279	708	236	255	8	TAATACTAT	HuA-CyV10 (KF726984)	82.2	*Podarcis filfollensis*
RI157/ITA	MZ201307	1,779	888	296	666	222	299	4	TAATACTAT	BaA-CyV2 (JF938079)	81.5	*Podarcis siculus*
RI175/ITA	MZ342901	1,773	669	223	837	279	271	4	TAGTATTAC	AntA-CyV^*c*^	77.3	*Podarcis siculus*
RI125/ITA	MZ201306	2,200	990	330	840	280	329	41	CAGTATTAC	RstoolA-CV^*c*^ (JF755410)	70.9	*Chalcides ocellatus*

a nt, nucleotides; aa, amino acids, HuA-CyV, human-associated cyclovirus; BaA-CyV, bat-associated cyclovirus; AntA-CyV, arboreal ant associated cyclovirus; RstoolA-CV, rodent stool-associated circovirus.

b https://talk.ictvonline.org/ictv-reports/ictv_online_report/ssdna-viruses/w/circoviridae, accessed on January 4, 2021.

c Genome sequence with the highest identity on interrogation of GenBank database with BLAST.

On genome sequence analysis, the CV strain RI125/ITA (accession MZ201306) shared a pairwise nt identity as high as 70.9% with a rodent stool-associated circular DNA virus (accession JF755410). The genome possessed two major ORFs, Rep, and Cap in opposite orientation. The 3′ downstream intergenic region included the stem-loop structure with the non-americ signal sequence, origin of replication (ori) (Table 1; Fig. 2). This genome structure resembled a type I genomic organization (15, 23) (Fig. 2). The virus segregated in a cluster of sequences generated from rodents (Fig. 4).

Strain RI175/ITA (accession MZ342901) shared 77.3% nt identity with the arboreal ant associated CyV 1 (accession MH545511), these viruses clustering together in the phylogenetic tree (42) (Fig. 3).

Both RI125/ITA and RI175/ITA were genome-wide below the species demarcation threshold (80% nt sequence identity at the genome level) adopted by International Committee on Taxonomy of Viruses (ICTV) for circoviruses and should be considered as putative novel species.

The CyV strain RI157/ITA (accession MZ201307), showed the highest nt identity (81.5%) to bat associated CyV 2 (accession JF938079), with which it grouped in the trees (Fig. 3).

The genome sequence of TN9-, TN12-, and TN25-like CyVs was also generated (Fig. 1). These groups of sequences accounted for 51.5% (*n* = 17/33) of the Rep-like sequences generated in the study. The TN9-like strain RI46/ITA (accession MZ201304) at the genome level exhibited the highest nt identity (83.6%) to human associated CyV 6, strain NG12 (accession GQ404854), with which it clustered in the trees (Fig. 3). Strain NG12 was identified in the stools of a non-polio acute flaccid paralysis (AFP) patient from Nigeria (21). In the partial rep-like sequence generated with the pan-circovirus nPCR, strain RI46/ITA showed the highest nt identity (97%) to CyV strain TN9, previously described in stools of a healthy child in contact with non-polio AFP patients in Tunisia (21). The complete CyV strain TN9 genome sequence was not available in public sequence databases (see GQ404906.1 for incomplete sequence).

The TN12-like strain RI196/ITA (accession MZ201308) was more similar (nt identity 82.2%) to human associated CyV 10, strain 7078A (KF726984) with which it grouped in the trees (Fig. 3). CyV strain 7078A has been found in respiratory samples of community-acquired pneumonia patients in Chile (54, 61). In the partial rep-like sequence generated with the pan-circovirus nPCR, strain RI196/ITA showed the highest nt identity (99%) to CyV TN12, identified in stools of a non-polio AFP patient in Tunisia (21). As observed for CyV strain TN9, the complete CyV strain TN12 genome sequence was also not available in public sequence databases (see GQ404905.1 for partial sequence).

Strain RI107/ITA (accession MZ201305) shared 95.9% nt identity with human associated CyV 4 (strain TN25, accession GQ404857), identified in stools of AFP patients of Tunisia (21). In the phylogenetic tree, this strain grouped with CyVs TN18 and TN25 (21).The genome was organized with two major ORFs, Rep and Cap. The CyV strain RI107/ITA presented a 169 nt putative intron in the Rep-encoding region, with donor (GT) and acceptor (AG) splice sites. Introns (~170 nt) have been identified in the Rep gene of other CyVs (21, 22) and, in general, are not uncommon in CyV genomes.

The genomes of the CyVs had 5′ intergenic region, located between the 5′ ends of the major ORFs, where the CyV non-anucleotide motif was located (Table 1; Fig. 2), at the apex of the potential stem-loop structure (7, 8). The 3′ intergenic region was 6 nt long for strain RI107/ITA and 8 nt long for RI46/ITA. Instead, the 3′ ends of Rep and Cap ORFs overlapped by 4 nt for strains RI157/ITA and RI175/ITA and by 8 nt for RI196/ITA (Table 1; Fig. 2).

## DISCUSSION

By screening a collection of samples obtained from hosts belonging to different Squamata species, CRESS DNA viruses were identified in about one third (31.7%, *n* = 33/104) of animals. Upon sequence analysis, a variety of CRESS DNA viruses were identified, many of which were similar to viruses associated with other animal species, including dogs, goats, birds, bats, and rodents. Most viruses were characterized as CyV (66.6%, *n* = 22/33), a taxon recently defined after the discovery of CRESS DNA viruses genetically related to CVs but with peculiar genetic features (7, 8).

CyVs were first reported in 2010 (21). Using consensus primers for the family *Circoviridae* (pan-circovirus PCR), the Rep sequences of CyV were detected in 7% to 17% stools from non-U.S. children with non-polio AFP, identifying 25 potentially different species from several countries, such Pakistan, Nigeria, and Tunisia (21). CyVs were identified in human stools and human-associated CyVs showed limited genetic overlap with those derived from consumed food (21). In 2013 CyV-Vietnam (CyV-VN) was detected in 4% of 642 cerebrospinal fluid (CSF) specimens of Vietnam patients with central nervous system (CNS) disease (27). CyV-VN was in parallel detected in the stools of healthy children, suggesting an oro-fecal or foodborne transmission. CyV-VN DNA was also detected in pigs and poultry suggesting the existence of animal reservoirs (27, 53, 56). In 2013, another CyV (CyV-VS5700009) was detected on screening of serum (15%) and CSF (10%) samples from Malawian patients with unexplained paraplegia (26). These findings quickly raised interest on CRESS DNA viruses with novel CyVs being identified from a variety of human samples and conditions (61, 62). The discovery of CyV in humans and its possible association with CNS disease elicited in 2013 a rapid risk assessment issued by the European Center for Disease Prevention and Control (ECDC) (53). The possible pathogenic role in the human host and the ecology of these viruses remain obscure, although several animal species could play a role as reservoirs of CyV for humans. Thus, far, based on the current ICTV classification criteria (a cut-off identity of 80% nt at the genome level), 12 distinct human-associated CyV species (HuACyV 1 to 12) have been recognized (7).

Epidemiological investigations and metagenomic studies have revealed the presence of CyVs in non-sterile samples from mammals (14, 20–28), birds (12, 21, 28–36), and insects (13, 17, 40–42). Several pieces of evidence suggest that CRESS DNA viruses are also common in reptiles. Metagenomic analysis and *in situ* RNA hybridization on liver and intestinal tissues allowed identifying CRESS DNA virus in the black-headed python *Aspidite melanocephalus* (43). Interestingly, genome fragments of CRESS DNA viruses have been found as endogenous viral elements (EVEs) inserted into the genome of the Speckled Rattlesnake *Crotalus mitchellii*, the King Cobra *Ophiophagus hannah*, and the Burmese Python *bivittatus* (44). Moreover, Rep-like sequences, regarded as CRESS DNA virus EVEs, have been detected in the Schneider’s Skink *Eumeces schneideri* and in the Ball Python, without a possible association with clinical signs (9).

In our study, Rep sequences of HuA-CyVs TN9 and TN12 were frequently identified in different geographic locations and reptile species. HuA-CyV TN9 was detected in *n* = 11/33 (33.4%) animals, including the Italian wall lizard and the Western Whip Snake while HuA-CyV TN12 was detected in *n* = 6/33 (18.2%) animals, including the Maltese Wall Lizard and the Ocellated Skink. An additional HuA-CyV, TN25-like, was identified from an Italian wall lizard. HuA-CyVs TN9, TN12, and TN25 were first identified in 2010 in stools of non-polio AFP patients (21), but TN9 and TN12 full genome sequence was not available. In order to rule out the detection of EVEs and to enlarge the information on genome diversity/organization of these viruses, the genome of HuA-CyV TN9, TN12, and TN25 identified in squamates were reconstructed (Fig. 2). On genome analysis, the TN9-like strain RI46/ITA was classified as HuA-CyV 6 (83.6% nt identity to strain NG12), while the TN12-like strain RI196/ITA was classified as HuA-CyV 10 (82.2% nt identity) and the TN25-like strain was classified as a HuA-CyV 4 (95.9% nt identity to strain TN25).

A total of *n* = 22 samples were collected from Linosa island Sicily, with *n* = 10/22 (45.5%) testing positive in our screening and *n* = 7/10 (70%) being characterized as human-associated TN9- and TN12-like CyVs. Because a collection of human sera of volunteers obtained from Linosa was available in our laboratory, we included these human sera in the molecular screening for CRESS DNA viruses. Enrollment of the volunteers in the study (63) was not based on specific inclusion criteria.

However, CyV DNA was not identified in the tested human sera. Likewise, on a screening on human sera in Central Italy, CyV DNA was not detected in *n* = 79 healthy individuals, while the prevalence rates were 1.6% (*n* = 1/60), 6.0% (*n* = 5/83) and 20.8% (*n* = 15/72) in transplant recipients, in patients with viral hepatitis and in HIV positive patients, respectively (55). This would suggest that sampling low-risk categories may markedly decrease the chances of detecting these viruses in the human host.

Because we tested the stools of the animals, we could not assess whether the identified viruses were able to infect and replicate actively in squamates or they were rather transported passively through the gastrointestinal tract, reflecting a dietary and/or environmental contamination. Indeed, phylogenetic analyses seem to suggest that CyVs are mainly arthropod infecting viruses (42, 64) and the result of arthropod contamination in food products (65). Interestingly, we also identified Rep sequences of possible arthropod origin in our sample collection, likely due to dietary habits of squamates. For one such virus, strain RI175/ITA, we generated the complete genome (Fig. 2) and the virus shared only 77.3% nt identity with arboreal ant associated CyV 1 (accession MH545511), thus representing a novel candidate CyV species.

Also, we identified a variety of CV sequences likely derived from other animal species (birds, dogs, bats, and rodents) and reflecting environmental pollution. We were able to reconstruct the genome sequences of CRESS DNA viruses, strains RI125/ITA and RI157/ITA, similar to a rodent-associated circular DNA virus (70.9% nt) and to a bat-associated CyV (81.5%), respectively. Strain RI125/ITA showed a type I genome organization (14, 23) and it was distantly related to other CVs identified in rodents (Fig. 2 and 4), qualifying as a novel unclassified CRESS DNA virus, while strain RI157/ITA was classified as a strain/variant of the bat-associated CyV 2 species (Fig. 2 and 3), based on the current ICTV classification criteria.

A limit of this study was the use of a pan-circovirus nPCR assay based on broadly reactive primers designed in 2010 to detect CRESS DNA viruses of the *Circoviridae* family (21). The design of consensus degenerated primers may generate a bias, as it relies on the sequence data available in the databases. However, in the span of a decade, sequence information on these viruses has dramatically increased. Metagenomic investigations using multiple strain displacement (MDA) protocol with phi29 DNA polymerase tend to select for circular DNA viruses, and can be used as a sequence-independent approach to generate information of CRESS DNA viruses in biological and environmental samples (66). Yet, this strategy is not as quick and costless as consensus PCRs for application in large-scale epidemiological investigations.

Squamates are now considered synanthropic in urban and peri-urban areas of the Mediterranean basin, and, based on our data, they could serve potential source of CyVs for the human host. The current COVID-19 pandemic has highlighted the threat to human health posed by animal viruses with zoonotic potential, and in general of human infections of animal origin. Virus surveillance should be reinforced in wild and domesticated animals in contact with humans, in order to continuously monitor and promptly characterize emerging and re-emerging zoonotic viruses, providing a baseline of the viral diversity and circulation, useful for tackling future infectious emergencies. Also, the identification of animal viruses closely related to human viruses provides useful information about their origin and ecology.

## MATERIALS AND METHODS

### Collection of samples from Squamata reptiles.

A total of 104 stool samples were collected from a population of different species of Squamata (*Podarcis siculus* (*n* = 66), *Podarcis filfolensis* (*n* = 6), *Hierophis carbonarius* (*n* = 2), *Tarentola mauritanica* (*n* = 13), *Testudo hermanii* (*n* = 2), *Chalcides ocellatus* (*n* = 12), and *Python regius* (*n* = 3)) during a study aiming to assess the role of reptiles as reservoirs of zoonotic parasites, such as *Leishmania*, carried out between 2020 and 2021 (67). In that study, reptiles were captured by lassoing or by hand, from urban/peri-urban areas in different regions of Italy, and transported to the Department of Veterinary Medicine, University of Bari. Samples were collected, following convenience sampling, from Apulia (*n* = 61), Sicily (*n* = 39), and Calabria (*n* = 4) (Table S1; Fig. S1).

Species of reptiles were identified using reference keys (68), and captured animals were then physically examined, to assess their health status. Data regarding species, biological state, sex, and region and area characteristics area in which animals were sampled, were recorded in each animal’s file. For each animal, fecal samples were collected and stored at −20°C.

All sampling procedures were conducted in accordance with the journal’s ethics policies as outlined on the journal’s author guidelines page, and the appropriate ethical review committee approval has been received. Protocols for lizard collection and sampling were authorized by the Ministry for Environment, Land and Sea Protection of Italy (approval number 0073267/2019), the *Societas Herpetologica Italica*, and the Istituto Superiore per la Protezione e la Ricerca Ambientale (approval number 71216).

### Collection of human sera.

From July 2020 to October 2020, serum samples of *n* = 101 volunteers from Linosa, Sicily were collected during a study on leishmaniosis (63), and subsequently sent to the Department of Veterinary Medicine, University of Bari (Italy). The study on humans was conducted in accordance with ethical principles (Declaration of Helsinki) and the research protocol was approved by the Ethical Committee of the University Hospital of Palermo, Italy (n. 8/2020). Written informed consent was obtained from each participant.

### DNA extraction from fecal and serum samples.

Faecal samples were homogenized in 10% Dulbecco’s modified Eagle’s medium (DMEM) and then centrifuged at 10,000 × *g* for 3 min. Nucleic acids were extracted from 200 μL of the supernatants using the QIAamp cador Pathogen minikit (Qiagen S.p.A., Milan, Italy), following the manufacturer’s protocol and stored at −80°C until use. An aliquot of 200 μL of each human serum sample was used for extraction of nucleic acids.

### Pancircovirus nPCR.

The nPCR protocol previously described (21) (Table S2), was used to detect CVs DNA. The 20-μL first-round PCR mixture was arranged using Platinum II Hot-Start Green PCR Master Mix (2X) (Invitrogen, Thermo Fisher Scientific). Briefly, the thermal protocol included a first step at 94°C × 2 min followed by 35 cycles of 94°C × 15 s, 52°C × 15 s, and 68°C × 15 s. One microliter of a 1:100 dilution of the PCR product was used in the second-round PCR using the same mix. The thermal protocol was the same as for the first-round PCR. To avoid cross-contamination, a strict separation between pre-amplification for reaction mix preparation, sample preparation and addition to mix, and amplification/post-amplification was adopted. DNA extracts were added to the mixtures in separate laminar flow cabinets. Liver samples homogenized from dogs with CV infections was used as positive controls, while distilled water were used as negative control.

### Genome amplification and sequencing.

All PCR positive products were sequenced directly. On the basis of the direct sequencing results, the full-length genome (Fig. 2), of a selection of CV-positive samples was generated. The circular DNA in selected samples was enriched by multiply primed rolling cycle amplification (RCA), using TempliPhi 100 amplification kit (GE Healthcare) and universal circovirus primer reverse (Table S2), according to the manufacturer’s instructions (69, 70).

A set of inverse nPCR primers (Table S2) was designed to recover the viral circular genome, amplifying a fragment of about 2 kb. Inverse nPCR primers were designed in a diagnostic universal genome fragment. The reverse and forward primers were designed with the 5′ end of the reverse facing the 5′ end of the forward primer. The design of primer pairs was based on sequencing results of the short diagnostic region obtained with degenerate primers. The primers were designed using the software Primer 3 implemented in Geneious version 10.2.4 (Biomatters Ltd., Auckland, New Zealand). The inverse PCR assays were performed with TaKaRa La *Taq* polymerase (TaKaRa Bio Europe S.A.S. Saint-Germain-en-Laye, France). The thermal protocol of inverse nPCR included a first step at 94°C × 2 min, followed by 35 cycles of 94°C × 30 s, 52°C × 30 s, and 68°C × 3 s, with a final extension of 68°C × 10 min. One microliter of a 1:100 dilution of the inverse PCR product was used in the second-round PCR using the same mix. The thermal protocol was the same as for the first-round PCR. The amplicons generated in the inverse PCR were directly sequenced. Sequence editing and annotation was done using Geneious version 10.2.4. software (Biomatters Ltd., Auckland, New Zealand).

### Sequence and phylogenetic analyses.

The purified PCR products with sufficient DNA concentration (>10 ng/μL) were directly sequenced in both directions by Eurofins Genomics (Ebersberg, Germany). The obtained sequences were aligned with cognate circovirus strains retrieved from the GenBank database by MAFFT algorithm (71). Phylogenetic analyses were performed with Bayesian inference by MrBaves software using four chains run for >1 million generations (72, 73) and Model Test software (http://evomics.org/resources/software/molecular-evolution-software/modeltest/) was used to identify the most appropriate model of evolution for the entire data set and for each gene individually. The identified program settings for all partitions, under the Akaike information criteria, included six-character states (general time reversible model) and a gamma distribution of rate variation across sites. Sequence editing, alignments, and phylogenetic analyses were performed by Geneious software version 9.1.8 (Biomatters, Auckland, New Zealand).

### Data analysis.

The categorical variables related to species, biological state, sex, and region in which animals were sampled were examined using Fisher exact test and χ^2^ test with Yates' correction, when appropriate. Statistical analyses were performed using the software R version 4.0.2 (https://www.R-project.org/) setting a statistical significance of *P*-value < 0.05.

### Data availability.

Nucleotide sequences of strains RI46/ITA, RI107/ITA, RI125/ITA, RI157/ITA, RI175/ITA, RI196/ITA retrieved in this study used for phylogeny were deposited in GenBank under accession nos. MZ201304, MZ201305, MZ201306, MZ201307, MZ342901, MZ201308, respectively.
