# Supplementary material for: Diversity of CRESS DNA Viruses in Squamates Recapitulates Hosts Dietary and Environmental Sources of Exposure
Source: Microbiol Spectr. 2022 May 26;10(3):e00780-22. doi: 10.1128/spectrum.00780-22 (PMC9241739; doi:10.1128/spectrum.00780-22)
Supplement: SUPPLEMENTAL FILE 1 — Supplemental material. Download spectrum.00780-22-s001.pdf, PDF file, 206 KB [file spectrum.00780-22-s001.pdf]

Supplemental Material

Diversity of CRESS DNA viruses in squamates recapitulates hosts dietary and environmental sources of exposure

Paolo Capozza, Gianvito Lanave, Georgia Diakoudi, Francesco Pellegrini, Roberta Cardone, Violetta Iris Vasinioti, Nicola Decaro, Gabriella Elia, Cristiana Catella, Alberto Alberti, Krisztián Bányai, Jairo Alfonso Mendoza-Roldan, Domenico Otranto, Canio Buonavoglia, Vito Martella*

* Corresponding author: Vito Martella,vito.martella@uniba.it

Table S1: Details (site, species) of the samples collected in the study.

Region	Collection site	Location	Species	Number of samples collected	nPCR* positive
Apulia	Site 1	Bari	*Hierophis carbonarius*	1	1
			Podarcis siculus	17	3
			Python regius	3	0
			Tarentola mauritanica	5	0
			Testudo hermanii	1	0
	Site 2	Brindisi	*Podarcis siculus*	14	8
			Tarentola mauritanica	1	1
	Site 3	Lecce	*Podarcis siculus*	17	4
			Tarentola mauritanica	2	1
Sicily	Site 4	Filicudi	*Podarcis siculus*	3	0
		Lipari	*Podarcis siculus*	5	5
		Malfa	*Podarcis siculus*	3	0
			Tarentola mauritanica	1	0
		Pollara	*Podarcis siculus*	1	0
			Tarentola mauritanica	1	0
		Salina	*Hierophis carbonarius*	1	0
		Vulcano	*Podarcis siculus*	2	0
	Site 5	Linosa	*Chalcides ocellatus*	12	7
			Podarcis filfolensis	6	3
			Tarentola mauritanica	3	0
			Testudo hermanii	1	0
Calabria	Site 6	Cassano All'Ionio	*Podarcis siculus*	4	0

*nPCR= Pancircovirus nested-PCR

Table S2: List of oligonucleotides used in this study.

Pathogen	Assay	Primers	Sequence (5'-3')	Amplification size (bp)	Reference
CV	Pan-CV PCR	763-CV-F1	GGI AYI CCI CAY YTI CAR GG	500	(1)
		764-CV-R1	AWC CAI CCR TAR AAR TCR TC		
	nPCR	765-CV-F2	GGI AYI CCI CAY YTI CAR GGI TT	400	(1)
		766-CV-R2	TGY TGY TCR TAI CCR TCC CAC CA		
Rodent-like CV	Inverse PCR	2041 208R	GTCCTCGAGCGTCTTCTTTG	>1500	this study
		2042 239F	GACTACGAGATCAGCCAGAT		
	nPCR	2043 78R	CTGTTGCTGAGCTTCTTGAG		
		2044 281F	CACTACAAGGGAATCAGGGA		
TN-9 like CyV	Inverse PCR	2045 131R	TATTCGGTAATTCCACGATGGTAT	>1500	this study
		2046 322F	AACTTGGGTGTACTATTATTGGGG		
	nPCR	2047 78R	AGTTGCTTGCTTTTGAACAGTATT		
		2048 379F	TAAGGAAGCTCAAGAAATTAACGC		
TN-12 like CyV	Inverse PCR	2059 162R	CTGGCTCAATCTAACACATCCTAT	>1500	this study
		2060 201F	GTGTAACTCGGCAATACGTTTAAT		
	nPCR	2061 117R	TGAAGTATCATACTACTGGGGGC		
		2062 305F	TTTCAAAGTATTCGCCCGATTTAG		
TN-25 like CyV	Inverse PCR	2079 351R	TTCTAGCTCCTTGAGTACTAGGTTC	>1500	this study
		2080 386F	AGGAGAGAGAAACATTAAAGCAGTG		
	nPCR	2081 309R	CGCCAGATTTAGAACAGTATGTCTT		
		2082 467F	TAGAATCTGTCATTCAAGTGCAGAA		
Bat-like CyV	Inverse PCR	2067 240R	AAACTCGGTTGCGATACTTGTAATC	>1500	this study
		2068 267F	AGGTATACGTGAACTCATGCAAGTA		
	nPCR	2069 105R	GTTATCTTCGTCTGATCCATTTGCC		
		2070 306F	ACCCAGAGATTTCAAAACCTACGTA		
Arborean ant-like CyV	Inverse PCR	2071 194R	GTTTTTATCCGCTACTTTCGTGGAA	>1500	this study
		2072 198F	GGCATTAATTTCAGCAAGTTTCTTT		
	nPCR	2073 66R	CCTACAAGAAGCTCAAGAACATGAC		
		2074 316F	CCTGCCTTTGAACAGTATTCTTTGT		

Legend: CV, circovirus; CyV, cyclovirus; Pan-CV, Pancircovirus; nPCR, nested PCR, R

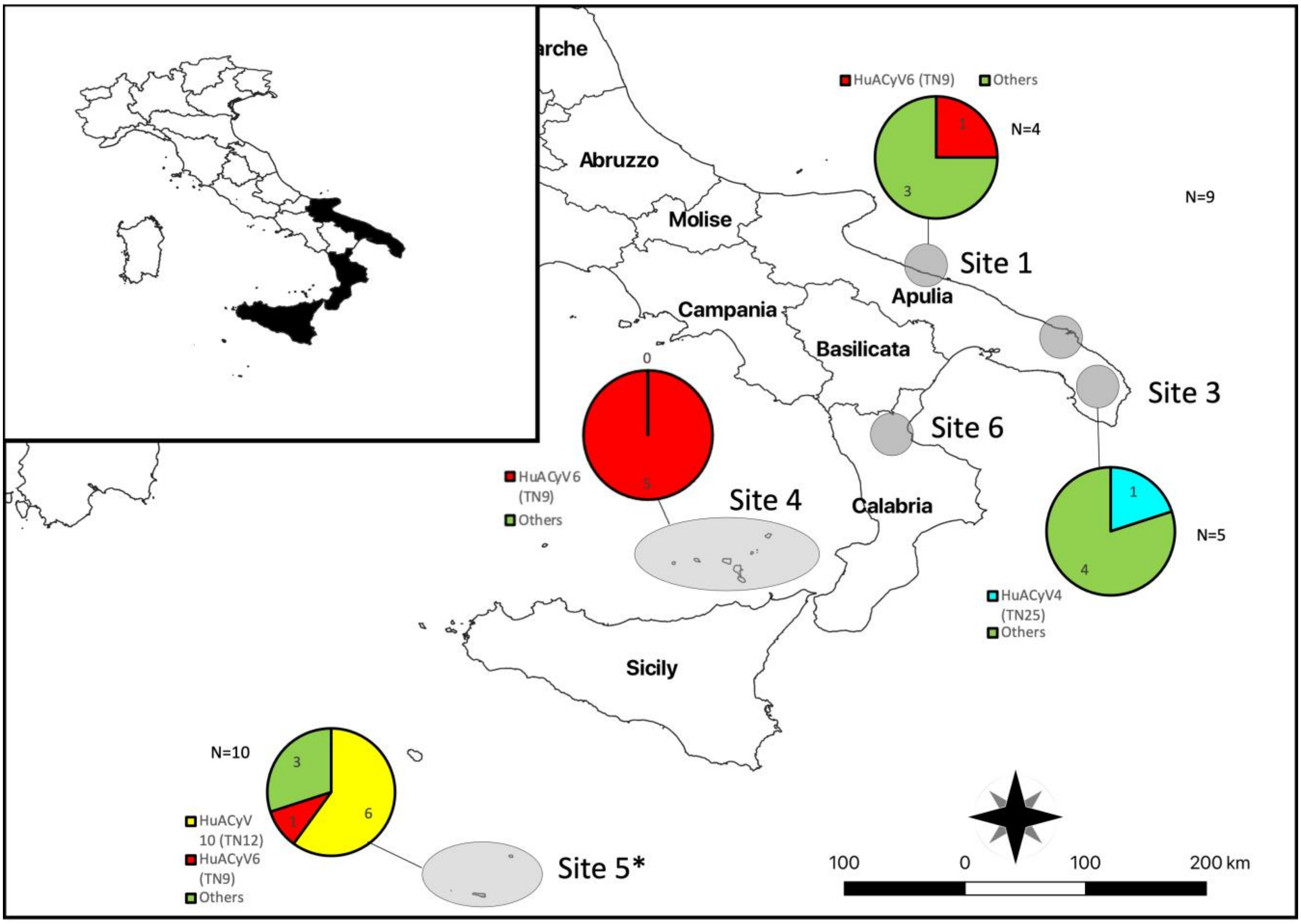

Supplementary figure 1: Italian regions (evidenced in black in the small window) and collection sites (in grey) of the animal samples screened in this study. For each site the number (N) of sample positive for CRESS DNA virus is indicated. The pie charts highlight the prevalence of human-associate cyclovirus (HuACyV) identified in reptiles in the study. The asterisk indicates the position of the site (Linosa) where human sera were sampled.

REFERENCES

1) Kapoor A, Mehta N, Esper F, Poljsak-Prijatelj M, Quan PL, Qaisar N, Delwart E, Lipkin WI. 2010. Identification and characterization of a new bocavirus species in gorillas. PLoS One 5:e11948.
